# Tissue-specific regulation of pregnane X receptor in cancer development and therapy

**DOI:** 10.1186/2045-3701-4-17

**Published:** 2014-04-01

**Authors:** Delira Robbins, Taosheng Chen

**Affiliations:** 1 Department of Chemical Biology and Therapeutics, St. Jude Children’s Research Hospital, 262 Danny Thomas Place, Memphis, TN 38105, USA

**Keywords:** Pregnane X receptor, Cancer, Nuclear receptors

## Abstract

As a ligand-dependent transcription factor of the nuclear hormone receptor superfamily, the pregnane X receptor (PXR) has a multitude of functions including regulating xenobiotic and cholesterol metabolism, energy homeostasis, gut mucosal defense, and cancer development. Whereas the detoxification functions of PXR have been widely studied and well established, the role of PXR in cancer has become controversial. With more than 60% of non-prescription and prescription drugs being metabolized by cytochrome P450 enzyme 3A4 (CYP3A4), a transcriptional target of PXR, insights into the regulation of PXR during systemic administration of novel treatment modalities will lead to a better understanding of PXR function in the context of human disease. Previous studies have suggested that PXR activation decreases drug sensitivity and augments chemoresistance in certain colon cancers mainly through the upregulation of CYP3A4 and multidrug resistance protein-1 (MDR1). Later studies suggest that downregulation of PXR expression may be oncogenic in hormone-dependent breast and endometrial cancers by reducing estrogen metabolism via CYP3A4; thus, higher estradiol concentrations contribute to carcinogenesis. These results suggest a differential role of PXR in tumor growth regulation dependent on tissue type and tumor microenvironment. Here, we will summarize the various mechanisms utilized by PXR to induce its diverse effects on cancerous tissues. Moreover, current approaches will be explored to evaluate the exploitation of PXR-mediated pathways as a novel mechanistic approach to cancer therapy.

## Introduction

Xenobiotic metabolism has been described as an adaptive response utilized by organisms exposed to drugs or environmental chemicals leading to induction of drug metabolizing enzymes to enhance water solubility and excretion [1]. Many compounds induce xenobiotic metabolism via the induction of Phase I (cytochrome P-450, or CYPs) and II enzymes, and Phase III transporters. Phase I enzymes include heme monooxygenases that catalyze hydroxylation or oxidation of hydrophobic compounds, resulting in enhanced water solubility [2]. CYPs are major Phase I enzymes and include CYP1A2, CYP2Cs, CYP2E1, CYP3A4, and CYP2B6 [3]. Phase II enzymes conjugate polar groups for detoxification and excretion that include glucuronide, glutathione and sulfate [3]. Phase III transporters are expressed in various tissues and are important in drug absorption, distribution, and excretion [4]. Phase III transporters include P-glycoprotein (P-gp; also known as multidrug resistance protein-1, or MDR1), multidrug resistance-associated proteins, and organic transporting polypeptides [4]. Nevertheless, induction of these drug metabolizing enzymes and transporters is regulated at the gene transcription level [1,4,5]. Orphan nuclear receptors are known to regulate bile acid [6], hormonal and lipid metabolism [7], hepatic steatosis [8], drug toxicity interactions [9], and cancer drug resistance via the induction of the previously mentioned metabolic enzymes [10]. The pregnane X receptor (PXR) is a key regulator of CYP3A gene expression [11]. Xenobiotics and steroids have been shown to regulate CYP3A gene expression via PXR activation [12]. Upon activation, PXR forms a heterodimer with retinoic acid receptor to bind to DNA [13]. Subsequently, PXR binds to response elements within the promoter regions of various transcriptional gene targets containing direct repeats of TGAACT spaced by three nucleotides (DR3) or four nucleotides (DR4) or an everted or inverted repeat of the TGAACT spaced by six nucleotides (ER6 and IR6) [11,13-19]. In addition, there are reports of ligand and promoter selective regulation of PXR-mediated gene expression [20]. In fact, different ligands of PXR can modulate PXR binding to different response elements in the promoter regions of targeted genes [20]. Moreover, co-activator interactions were ligand-dependent via differences in the conformations of the PXR-ligand complexes [20]. For example, the anticancer drugs paclitaxel and cisplatin induced PXR binding, more strongly to response elements within the MDR1 promoter than those within the CYP3A4 promoter [20]. Interestingly, among various diverse species, there is approximately 95% sequence homology in the DNA binding domain (DBD) of PXR but only approximately 80% homology in the ligand binding domain (LBD). Previous studies have revealed significant interspecies differences in PXR activation profiles between human PXR (hPXR) and mouse PXR (mPXR) [11]. For example, known PXR ligands such as rifampicin, clotrimazole, lovastatin, and phenobarbital were efficacious activators of hPXR, as evidenced by increased *CYP3A* gene expression, but not mPXR. On the other hand, pregnenolone 16α-carbonitrile was a potent inducer of mPXR but not hPXR [11]. Initially, there were several lines of evidence that suggested high tissue-specific expression of PXR in liver and intestine [3,11,14,21]. However, further studies revealed PXR expression in mouse kidney [14], ovary and uterus [22] and in human brain [23] and breast tissues [24]. It was later extended by Lamba and colleagues to include the expression of PXR in stomach, adrenal glands and bone marrow of human tissue samples [25]. Clinical relevance of PXR expression is continuing to emerge and suggest PXR is a potential therapeutic target to decrease tumor growth. PXR is highly expressed in certain cancers [26-30], promoting cell proliferation and chemoresistance [31-33], and potentially contributing to malignancy [33] in both preclinical models and clinical patient samples. Interestingly, PXR overexpression by stable transfection of hPXR or by pharmacologic activation has been shown to protect cells from apoptosis in HepG2 liver hepatocellular carcinoma [34]. Similar results were seen in HCT116 human colon cancer and LS180 intestinal human colon adenocarcinoma cells by overexpressing constitutively activated PXR or through pharmacologic activation of PXR with the cognate ligand rifampicin [35], suggesting an anti-apoptotic role of PXR in carcinogenesis. On the other hand, PXR has been shown to be a regulator of apoptosis in tissues that are outside of the metabolic realm of the liver and intestine, including tumor tissues within endometrial and breast cancers [36,37]. Thus, these findings suggest novel tissue-specific functions of PXR that warrant further analysis as a potential target of chemotherapeutic utility. Moreover, co-activator versus co-repressor expression, tumor microenvironment, and differential ligand expression have all been shown to play key roles in the tissue-specific functions of PXR [38]. With PXR regulating a multitude of diverse functions to maintain cellular homeostasis, targeting PXR clinically becomes a promising, yet difficult task. Here, we focus on the differential regulation of PXR in a tissue-specific manner and how the tissue-specificity of expression/function may affect the overall outcome of targeting PXR in human disease. Table 1 summarizes the tissue-specific features of PXR in cancer development.

**Table 1:** Tissue-specific characteristics of PXR in cancer development

	**Characteristics of PXR activation in cancer development**			
**Cancer type**	**Pro-proliferative**	**Anti-apoptotic**	**Pro-apoptotic**	**Anti-proliferative**
Ovarian carcinoma	Yes [33]			
Colon cancer	Yes [48]	Yes [35]		Yes [51]
Breast carcinoma	Yes [26]		Yes [37]	
Hepatoma cells		Yes [34]		
Prostate cancer		Yes [32]		
Endometrial cancer		Yes [36]		

### Pro-proliferative and anti-apoptotic properties of PXR

The role of PXR in cancer has received considerable attention due to its clinical relevance and potential contribution to the “malignant” phenotype of cancers. Gupta and colleagues [33] found that PXR was expressed in human ovarian carcinoma cells (OVCAR8 and SKOV-3), promoting cell proliferation and drug resistance through rifampicin-mediated PXR induction of target genes. In addition, *in vivo* data utilizing SKOV-3 xenografts suggested that cognate ligand activation of PXR promoted tumor growth by significantly inactivating and decreasing cytotoxic drug concentrations via upregulation of PXR-mediated drug-metabolizing enzymes CYP2B6, CYP3A4 and UDP glucuronosyltransferase 1A1 (UGT1A1) [33]. Similar anti-apoptotic results were translational in both normal mouse epithelium and human colon cancer cells [35]. Interestingly, stable viral transduction of the constitutively active viral protein 16 activation domain fused to the amino terminus of human PXR (VP-PXR) or pharmacologic activation via rifampicin treatment protected cells from deoxycholic acid-induced apoptosis in colon cancer cells, a mechanistic effect outside of the canonical PXR xenobiotic function [35]. Furthermore, PXR overexpression promoted induction of anti-apoptotic genes, *BAG3, BIRC2,* and *MCL-1* and downregulated pro-apoptotic genes *BAK-1* and *TP53* through both genetic (using constitutively active VP-PXR) and pharmacologic (via rifampicin) activation of PXR [35]. These reports stress the importance of PXR activation in the biology of human cancers.

With chemoresistance being a significant barrier to the efficacy of chemotherapeutic drugs, understanding how PXR may regulate cell proliferation, chemoresistance, and tumorigenesis is needed to identify novel targets for cancer therapeutic drug discovery and development. It has been well established that PXR is efficiently activated by several steroid hormones, including estrogen, as indicated by the induction of the CYP3A family of steroid hydroxylases in both *in vivo* models and patient samples [11,14,15,28,39-42]. PXR is expressed in reproductive uterine and ovarian tissues, and PXR transcriptional targets CYP3A4 and CYP3A7 play roles in steroid metabolism in human endometrium [22,43]. Stronger nuclear staining of PXR has been reported in samples from endometrial cancer patients than in normal patient endometrium samples. Interestingly, Masuyama and colleagues [28] reported a significant inverse relationship between PXR expression and estrogen receptor-α (ER-α) status in endometrial cancer tissues. Their findings suggested higher PXR and CYP3A4/7 expression in endometrial cancer tissues with lower ER-α status [28]. Masuyama and colleagues [28] also suggested that PXR-CYP3A4/7 signaling may serve as an oncogenic alternative pathway that contributes to carcinogenesis in endometrial cancer tissues with low ER-α status. In other hormone-dependent neoplasms such as prostate cancer, Chen and colleagues [32] found that PXR activation by the selective potent agonist SR12813 enhanced resistance to chemotherapeutic drugs taxol and vinblastine via PXR-mediated upregulation of CYP3A4 and MDR1 in human prostate cancer PC3 cells. Furthermore, PXR knockdown using shRNA constructs enhanced the chemosensitivity of prostate cancer cells to chemotherapeutic drugs, suggesting a contributing role of PXR to chemoresistance in prostate cancer [32].

Additional studies suggest that the pro-proliferative and anti-apoptotic functions of PXR are tumor-specific. Fibroblast growth factor 19 (FGF19) promotes liver carcinogenesis, and overexpression of the FGF receptor 1–4 strongly correlates with neoplastic transformation in various cancers [44-47]. Rifampicin-mediated PXR activation can significantly enhance cell proliferation and tumor invasiveness [48]. A previous study showed that the PXR-mediated cell proliferative and metastatic phenotype was promoted by FGF19 expression both *in vitro* and *in vivo*[48]. Furthermore, PXR can directly bind to the promoter region of the *FGF19* gene via DR3 and ER6 elements in both cancer and normal intestinal crypt cells. However, FGF19 signaling was only induced in colon cancer tissue, suggesting a PXR tumor specific mechanism of proliferation [48]. Table 2 summarizes the tissue-specific pro-proliferative and anti-apoptotic functions of PXR; in these studies, the activity of PXR was enhanced by PXR overexpression or pharmacologic activation through the use of PXR ligands, or reduced by PXR knockdown using small interfering RNA.

**Table 2:** Pro-proliferative and anti-apoptotic functions of PXR

**Pro-proliferative functions of PXR**		
**Tissue specification**	**PXR activation approach**	**References**
Ovarian carcinoma	PXR activation by cognate ligands induced cell proliferation and drug resistance. In SKOV-3 xenografts, PXR ligand activation induced cell proliferation and tumor growth.	[33]
Colon carcinoma	Activation of PXR enhanced cell growth, invasion, and metastasis in human colon tumor cell lines and human colon cancer xenograft models via PXR-mediated FGF19 signaling.	[48]
Breast carcinoma	Immunohistochemistry, quantitative reverse transcriptase PCR, and microarray analysis all revealed PXR expression in carcinoma tissues but not in nonneoplastic or stromal cells in breast carcinoma patient samples. In breast carcinoma cells, pharmacologic activation of PXR via rifampicin increased PXR target genes (OATP1A2 and CYP3A4). Positive correlation between PXR, OATP1A2, and increasing tumor grade in breast carcinoma patient samples.	[26]
**Anti-apoptotic functions of PXR**		
**Tissue specification**	**PXR activation approach**	**References**
Colon cancer	PXR activation via a genetic approach (constitutive activation) or pharmacologic activation via rifampicin protected colon cancer cells from chemically induced apoptosis. PXR activation in transgenic mice inhibited bile acid-induced colonic epithelial apoptosis and sensitized mice to dimethylhydrazine-induced colon carcinogenesis.	[35]
Hepatocytes/Hepatoma cells	Agonists of PXR increased hepatocyte viability and protected them from staurosporine-induced apoptosis via the induction of Bcl-2 and Bcl-xl in human and rat hepatocytes. PXR agonists protected HepG2 human hepatoma cells from Fas-induced apoptosis via Bcl-2 and Bcl-xl induction.	[34]
Prostate cancer	PXR activation via a potent selective PXR agonist, SR12813, increased resistance to chemotherapeutics paclitaxel and vinblastine. Knockdown of PXR via shRNA decreased resistance and increased sensitivities to chemotherapeutics.	[32]
Endometrial cancer	PXR overexpression caused significant decreases in apoptosis in the presence of paclitaxel or cisplatin. PXR downregulation enhanced apoptosis in the presence of paclitaxel, cisplatin, and medroxyprogesterone acetate.	[36]

### Pro-apoptotic and anti-proliferative properties of PXR

Apoptosis is a complex process involving various signaling pathways and is both environment- and receptor/ligand-dependent. Interestingly, PXR has received attention as a novel mediator of apoptosis via both p53-dependent and -independent pathways. PXR expression is significantly lower in colon cancer cell lines such as HCT116 and HT29 [49]. Ectopic expression of human PXR via stable transfection significantly inhibited anchorage-independent growth and cell proliferation both *in vitro* and *in vivo* suggesting a novel physiological role of PXR in protecting the cells from unregulated oncogenic proliferation [50]. Moreover, p21 (WAF1/CIPI) expression was significantly elevated, and G_0_/G_1_ cell cycle arrest was induced by PXR. Therefore, the question remains: Can PXR regulate p53-mediated tumor suppression? Verma and colleagues [37] reported that various structurally and functionally distinct PXR activators inhibited cell proliferation in breast cancer cell lines MCF-7 and ZR-75-1, both containing wild-type p53. In addition, proliferative inhibition was induced by cell cycle arrest at the G_1_/S phase followed by apoptosis [37]. When they investigated which genes were responsible for decreased growth, they found the pro-apoptotic genes, *CDKN1A* (p21), *BBC3* (PUMA) and *Bax* were all upregulated. This study suggested that wild-type p53 was mechanistically required for the anti-proliferative phenotype of PXR. Moreover, PXR activators increased nitric oxide in these cells via iNOS. To determine the biological significance of PXR levels, PXR was knocked down via siRNA, which reduced iNOS levels, decreasing apoptosis, suggesting a PXR-mediated mechanism of apoptosis in breast cancer cells. However, the role of PXR activation in breast cancer cells remains controversial. Similar to the aforementioned study, overexpression of PXR sensitized cells to paraquat-induced oxidative stress when treated with a PXR agonist [51]. The dependency of the anti-proliferative effect of PXR on wild-type p53 suggested a functional relationship between PXR and p53 signaling. Interestingly, a recent report showed that p53 interacts with and inhibits the function of PXR [52]. However, whether the functional interaction between PXR and p53 leads to a mutual inhibitory effect, whether the PXR-p53 interaction is tissue specific, and how stimulants of each pathway affect the interaction and its biological significance still remain elusive. Table 3 summarizes the tissue-specific pro-apoptotic and anti-proliferative functions of PXR; in these studies, the activity of PXR was induced either by constitutive activation or pharmacologically by using ligands of PXR.

**Table 3:** Anti-proliferative and pro-apoptotic functions of PXR

**Anti-proliferative functions of PXR**		
**Tissue specification**	**PXR activation approach**	**References**
Colon cancer	Ectopic expression of PXR significantly inhibited anchorage-independent growth and cell proliferation *in vitro* and xenograft tumor growth *in vivo* via G_0_/G_1_ cell-cycle arrest.	[50]
Liver and colon carcinogenic tissue	PXR overexpression sensitized cells to paraquat-induced oxidative stress when treated with PXR agonists.	[51]
**Pro-apoptotic functions of PXR**		
**Tissue specification**	**PXR activation approach**	**Reference**
Breast cancer	SXR activators inhibited cell proliferation and induced apoptosis in breast cancer cells. Wild-type p53 was mechanistically required for the anti-proliferative phenotype of PXR.	[37]

### Regulation of PXR under different tissue and cellular contexts

PXR is expressed mainly in liver, intestine, and colon tissues [39]. However, PXR activation has been shown to be tissue-specific, based on differential ligand availability and intratumoral concentrations of endogenous steroid hormones, which may also be PXR activators within certain tissues. The relationship between ligand availability and PXR activation has been found to modulate estrogen accumulation and disease progression in hormone-responsive neoplasms, such as breast and endometrial cancers. For example, the positive correlation between estrogen and breast cancer progression has been well established [53]. However, the role of PXR activation has recently received considerable attention [26]. The organic anion transporter polypeptide 1A2 (OATP1A2) is a transporter of hormone conjugates, facilitating the cellular uptake of hormones [54]. Initially, Miki and colleagues [26] found the expression of both PXR and OATP1A2 in human breast carcinoma. Moreover, their findings suggested that PXR and OATP1A2 were potential markers of dedifferentiation and disease progression. Later, Meyer zu Schwabedissen and colleagues [55] showed a direct relationship between the pathogenesis of hormone-responsive breast cancer and increased estrogen accumulation via PXR mediated upregulation of OATP1A2. Estrogen, particularly its intratumoral production, contributes to the pathogenesis of endometrial cancer. Aromatase converts androgen to estrone and is expressed at higher levels in neoplastic endometrium than in normal tissues [56-58]. Similarly, estrone sulfatase converts estrone sulfate (E_1_S) into estrone and is found in higher levels in endometrial cancer than in normal tissues [59]. Mechanistically, the activities of both aromatase and estrone sulfatase serve as an active source of biologically active estrogen within endometrial carcinoma tissues [56-59]. Interestingly, an earlier study showed that OATP1A2 upregulation was tumor-specific and mediated by PXR activation, resulting in enhanced uptake of E_1_S, an estrogen metabolite, and upregulation of estrogen-targeted genes [55]. Pharmacologically, utilizing the PXR inhibitor A-792611, rifampicin-induced estrogen receptor activity in estrogen receptor-positive T47-D cells was significantly reduced, suggesting the mechanistic feasibility of PXR antagonists as therapeutic agents for breast cancer [55].

PXR is activated by various pharmaceutical agents, however whether and how various metabolites of the parental compound affect PXR activity can lead to differential effects. In addition, variants of the parental compounds may regulate PXR activation in a tissue specific manner. For example, vitamin E is known to exist in various isoforms of either tocotrienols or tocopherols. Zhou and colleagues [60] showed that isoforms of vitamin E, known PXR activators, can regulate PXR target genes in a tissue-specific manner. Target genes such as *CYP3A4*, *UGT1A1*, and *MDR1* were all induced in primary hepatocytes; however, *CYP3A4* could not be induced in intestinal LS180 cells. Furthermore, nuclear receptor co-repressor expression was higher in LS180 cells, suggesting that differential expression of co-factors can contribute to alternative modes of activation in a tissue specific manner [60].

Similar results have been seen in previous studies concerning the importance of transcriptional co-activators and co-repressors in regulating nuclear receptor activation [61]. Nuclear receptor co-repressors and co-activators are common and are shared among all nuclear receptors. The p160 steroid receptor coactivator (SRC) family consists of three members, SRC-1, SRC-2, and SRC-3. These co-activators harbor acetyltransferase activity and bind to nuclear receptors to enhance their activity [62]. The SRC family has been well-studied and is amplified or overexpressed in certain cancers. Misiti and colleagues [38] found despite ubiquitous expression, nuclear receptor co-activators and co-repressors are expressed at different levels in a tissue-specific manner and therefore differentially affect nuclear receptor-mediated gene activation and hormonal regulation. Nevertheless, PXR expression is elevated in several human cancers including colon, breast, prostate, intestinal, esophageal, endometrial, and ovarian. Thus, differential tissue expression of not only PXR but of co-activators and co-repressors in human cancer makes generalized treatment approaches a trend of the past and personalized gene-based therapeutics a promising treatment modality of the future.

### Clinical relevance

PXR plays a pivotal role in the development of multidrug resistance via the induction of drug metabolizing enzymes and transporters that mediate metabolism, detoxification, and elimination of most pharmaceutical agents at clinically relevant concentrations. CYP3A4 is an important mediator of drug metabolism and is transcriptionally regulated by PXR. PXR also regulates drug efflux by inducing the expression of MDR1. This has applications in various steroid-dependent neoplasms, such as breast and endometrial cancers that express higher levels of PXR in neoplastic tissues than in normal tissues [26,28]. Moreover, Miyoshi and colleagues [63] suggest using intratumoral levels of CYP3A4/7 mRNA as predictors of response to antineoplastic drugs such as docetaxel in the treatment of breast cancer; and further involvement of the CYP3A family in drug clearance may have subsequent effects on patient prognosis [28]. Thus, a better understanding of the mechanisms that underlie the involvement of PXR in regulating steroid metabolism and the intratumoral steroid levels involved in the carcinogenesis of hormone-dependent cancers, as well as PXR-mediated multidrug resistance, is needed. However, the ability of PXR to promiscuously bind to various structurally diverse compounds may cause targeting ligand-mediated PXR activation to be a promising yet intricate task to complete. When designing drugs to target PXR-mediated activation, it is important to take into account the molecular network of PXR with other cellular proteins and signaling pathways, including the molecular cross-talk of PXR with other nuclear receptors such as constitutive androstane receptor (CAR) that bind to similar response elements and activate an overlapping set of genes. Although concurrent administration of PXR antagonists with traditional chemotherapeutic drugs may circumvent drug-drug interactions and toxicities, the consideration of documented bone demineralization, unanticipated hypersensitiveness and toxicities associated with such combinations must not be disregarded.

Paclitaxel is a traditional chemotherapeutic known to activate PXR and enhance MDR1-mediated drug clearance. Interestingly, docetaxel, a related antineoplastic agent, does not activate PXR and mechanistically cannot displace transcriptional corepressors from PXR. This suggests the importance of modulating PXR activity as a mechanism to regulate drug metabolism and efficiency as well as clearance. Nonetheless, previous studies have assessed the clinical application of PXR modulators to sensitize cells to chemotherapeutic drugs. Some of the reported PXR antagonists include ketoconazole, enilconazole, HIV protease inhibitor A-792611, coumestrol, and FLB-12.

#### Ketoconazole

Ketoconazole [64], a PXR antagonist first described by Takeshita and colleagues [65], disrupts the binding of co-regulators, both activators and repressors, to the surface of PXR in an agonist-dependent manner. This compound has been shown to initiate non-competitive inhibition with PXR agonists by inhibiting PXR’s association with its co-activator SRC-1 [64-67]. Moreover, Wang and colleagues showed the importance of the AF-2 region located outside of the PXR LBD in the mechanistic inhibition of PXR by ketoconazole. However, ketoconazole has pleiotropic effects on cellular targets and unpredictable kinetics [68-72]. Thus, more target-specific drugs are needed to antagonize PXR activation.

#### Enilconazole

Enilconazole [67], a ketoconazole derivative, has significant inhibitory effects on rifampicin-activated human PXR in the presence of paclitaxel. It has been considered an “activating antagonist” due to its ability to modestly induce activation of PXR alone, but it has been shown to significantly inhibit PXR in the presence of a PXR agonist.

#### HIV protease inhibitor A-792611

Some HIV protease inhibitors are also potent CYP3A4 inhibitors; however, like enilconazole, these compounds can also induce PXR activation, yet the inhibitory effects on CYP3A4 activity outweigh the agonistic effects on PXR. Examples of such compounds include HIV protease inhibitors such as ritonavir [73]. The novel HIV protease inhibitor (s)-1-[(1 S, 3S, 4S)-4-[(S)-2-(3-benzyl-2-oxo-imidazolidin-1-yl)-3,3-dimethyl-butyrylamino]-3-hydroxy-5-phenyl-1-(4-pyridin-2-yl-benzyl)-pentylcarbamoyl]-2,2-dimethyl-propyl-carbamic acid methyl ester (A-792611) is mainly metabolized by CYP3A4 but is also an inhibitor of CYP3A4 activity [74]. Healan-Greenberg [74] reported target specific effects of A-792611 on PXR and no effects on other nuclear receptors that regulate P450s such as CAR and farnesoid X receptor. Thus, A-792611 was reported to be a functional antagonist of PXR in human but not rat hepatocytes [74].

#### Coumestrol

Coumestrol [75], a naturally occurring phytoestrogen, is a member of the isoflavonoid family. Wang and colleagues [75] demonstrated that coumestrol suppressed SR12813-mediated PXR induction of CYP3A4 and CYP2B6 in primary human hepatocytes. Furthermore, the naturally occurring phytoestrogen antagonized co-regulator recruitment with a mechanism similar to that of ketoconazole, binding outside the ligand binding pocket. Ekins and colleagues [76] utilized computational models to suggest that coumestrol could bind at the AF-2 domain of PXR. However, poor solubility of coumestrol is a disadvantage [75]. Therefore, the clinical use of coumestrol requires further investigation.

#### FLB-12

Venkatesh and colleagues [77] successfully characterized and developed a novel azole analogue [1-(4-(4-(((2R,4S)-2-(2,4-difluorophenyl)-2-methyl-1,3-dioxolan-4-yl)methoxy)phenyl)piperazin-1-yl)ethanone (FLB-12)]. Similar to those of A-729611, the targeted effects of FLB-12 are PXR-specific, with no effects on other orphan nuclear receptors [77]. Interestingly, they reported that the small-molecule FLB-12 was significantly less toxic than other PXR antagonists such as ketoconazole; and decreased acetaminophen hepatotoxicity *in vivo*. Likewise, FLB-12 induced additional effects by preventing resistance to 7-ethyl-10-hydroxycamptothecin (SN-38) in colon cancer cells [77]. Thus, this class of compounds encourages the design and development of PXR-targeted antagonists that can inhibit drug resistance, with limited toxicity, to enhance chemosensitivity and therapeutic efficacy with clinical significance.

## Conclusions

Overall, there are two paradigms of signaling to be targeted in regards to PXR activation 1) PXR-mediated signaling in drug metabolism and 2) PXR-mediated signaling in cell proliferation, apoptosis and tumor aggressiveness, which opens numerous avenues for targeted therapeutic application. Therefore, identifying significant biomarkers within the dichotomy of PXR signaling could optimize strategies for designing potential PXR antagonists with tissue-specific, desirable outcomes. Nevertheless, the therapeutic outcome of a drug depends on multiple variables. However, in regard to PXR, differential tissue-specific expression of the protein, and its co-regulators may play a significant role in drug bioavailability in non-neoplastic versus neoplastic tissues, as well as modulation of the drug’s mechanism of action at tumor sites. Together, taking into consideration these aspects of PXR tissue specific regulation, and identifying novel small molecules that produce the desired effect with limited toxicities will lead to the design and characterization of PXR-targeted small molecules with significant tissue-specific, pharmacologic properties.

## Abbreviations

AF-2: Activation function-2; BAG3: BCL2-associated anthanogene 3; BBC3: BCL-2 binding component 3; BIRC2: Baculoviral IAP repeat containing 2; CAR: Constitutive androstane receptor; CDKN1A: Cyclin-dependent kinase inhibitor 1A; CYP: Cytochrome P450 enzyme; CYP3A: Cytochrome P450 enzyme 3A; DBD: DNA binding domain; E1S: Estrone sulfate; ER-α: Estrogen receptor – alpha; FLB-12: [1-(4-(4-(((2R,4S)-2-(2,4-difluorophenyl)-2-methyl-1,3-dioxolan-4-yl)methoxy)phenyl)piperazin-1-yl)ethanone; FGF19: Fibroblast growth factor 19; HIV: Human immunodeficiency virus; iNOS: Inducible nitric oxide synthase; LBD: Ligand binding domain; MCL-1: Myeloid cell leukemia sequence 1; MDR1: Multidrug resistance protein 1; OATP1A2: Organic anion transporter polypeptide 1A2; PXR: Pregnane X receptor; SN-38: 7-ethyl-10-hydroxycamptothecin; SRC: Steroid receptor coactivator; siRNA: Short interference RNA; TP53: Tumor protein p53; UGT: UDP glucuronosyltransferase; VP-PXR: Viral protein 16 activation domain fused to the amino terminus of human PXR.

## Competing interests

The authors declare that they have no competing interest.

## Authors’ contributions

DR drafted the manuscript. TC participated in the coordination and helped to draft and finalize the manuscript. Both authors have read and approved the final manuscript.
